# From Neuroadaptation to Neuroprogression: Rethinking Chronic Cocaine Exposure Through a Model of Cocaine-Related Cerebropathy

**DOI:** 10.3390/jcm15062222

**Published:** 2026-03-14

**Authors:** Manuel Glauco Carbone, Icro Maremmani, Filippo Della Rocca, Giulia Gastaldello, Luca Mazzetto, Alessandro Bellini, Roberta Rizzato, Rossella Miccichè, Beniamino Tripodi, Claudia Tagliarini, Maurice Dematteis, Angelo Giovanni Icro Maremmani

**Affiliations:** 1 Division of Psychiatry, Department of Medicine and Surgery, University of Insubria, Viale Luigi Borri 57, 21100 Varese, Italy; manuelglaucocarbone@gmail.com (M.G.C.); ggastaldello@studenti.uninsubria.it (G.G.); lmazzetto@studenti.uninsubria.it (L.M.); alessandro.bellini1992@gmail.com (A.B.); beniamino.tripodi90@gmail.com (B.T.); 2 VP Dole Research Group, G. De Lisio Institute of Behavioural Sciences, Via di Pratale, 3, 56121 Pisa, Italy; filippo.dellarocca@yahoo.it (F.D.R.); angelogiovanniicro.maremmani@unicamillus.org (A.G.I.M.); 3 Department Faculty of Medicine, Saint Camillus International University of Health Sciences, Via di Sant’Alessandro 8, 00131 Rome, Italy; 4 Addiction Unit, Department of Mental Health and Addictions, ASL 5 Liguria NHS, Via Dalmazia 1, 19124 La Spezia, Italy; 5 Psychiatry Unit, Department of Mental Health, Addiction Prevention and Treatment, ASST Sette Laghi—Ospedale di Circolo e Fondazione Macchi, Viale Luigi Borri 57, 21100 Varese, Italy; roberta-rizzato@libero.it (R.R.); rossella.micciche@asst-settelaghi.it (R.M.); 6 Division of Psychiatry, Department of Mental Health and Addictions, ASST Crema, Via Largo Ugo Dossena 2, 26013 Crema, Italy; 7 Psychiatric Diagnosis and Treatment Service, Department of Mental Health, Sant’Elia Hospital, ASP 2 Caltanissetta, 93100 Caltanissetta, Italy; claudiatagliarini8@gmail.com; 8 Department of Pharmacology and Addiction Medicine, Faculty of Medicine, Grenoble Alpes University Hospital, Grenoble Institute of Neurosciences, Université Grenoble Alpes, INSERM U1216, Site Santé—Allée des Alpes, 38000 Grenoble, France; mdematteis@chu-grenoble.fr

**Keywords:** cocaine-specific cerebropathy, neurodegeneration, neuroprogression, dopaminergic dysregulation, neurodevelopmental vulnerability

## Abstract

**Background:** Chronic cocaine exposure is increasingly associated with persistent brain alterations, yet it remains unclear whether these changes reflect reversible neuroadaptation, accelerated brain ageing, or a degeneration-like trajectory in a vulnerable subgroup. This Perspective proposes a neuroprogressive vulnerability framework—referred to as *cocaine-specific encephalopathy/cerebropathy* only in a heuristic sense—to organise heterogeneous evidence without implying a distinct neurodegenerative disease entity. **Methods:** We conducted a structured, critical synthesis of peer-reviewed human and preclinical literature (PubMed, Scopus, Web of Science; inception to December 2025), integrating neuroimaging (MRI/DTI/fMRI/PET/SPECT), neuropathology/post-mortem findings, neurochemical and molecular mechanisms, and neuropsychological outcomes, with explicit attention to confounders (polysubstance use, psychiatric and medical comorbidity, HIV, vascular risk, abstinence duration). **Results:** Convergent evidence supports a multi-hit vulnerability model in which chronic stimulant exposure may weaken neural resilience through dopaminergic dysregulation, oxidative stress, mitochondrial dysfunction, neuroinflammatory signalling, and putative α-synuclein–related mechanisms. Human imaging studies consistently implicate fronto–striato–limbic circuits and suggest possible cerebellar involvement, but findings are heterogeneous and often cross-sectional; direct evidence of progressive neuronal loss or disease-defining proteinopathies attributable to cocaine remains limited. **Conclusions:** Rather than asserting cocaine-induced classic neurodegeneration, we outline an exploratory framework in which chronic cocaine exposure may increase susceptibility to neuroprogressive impairment in a subset of biologically vulnerable individuals. Longitudinal multimodal studies combining advanced imaging, biomarkers, and phenotypic stratification are needed to clarify causality, temporal progression, and reversibility with sustained abstinence.

## 1. Introduction

Chronic cocaine exposure has traditionally been studied from the perspectives of addiction neuroscience, acute toxicity, and psychiatric comorbidity. Yet converging evidence from neuroimaging, molecular research, and clinical observation raises a broader—and scientifically provocative—question: could long-term cocaine use contribute to neuroprogressive patterns that resemble, anticipate, or interact with recognised neurodegenerative mechanisms?

This perspective does not propose a new diagnosis. Instead, it examines whether chronic stimulant exposure might heighten pre-existing neurobiological vulnerabilities and weaken neural resilience, potentially leading to a trajectory tentatively termed cocaine-specific encephalopathy. The ensuing discussion synthesises current evidence with explicit methodological caution, integrating neurochemical, neuropathophysiological, and neuroanatomical observations into a heuristic framework to guide future empirical research.

Cocaine use disorder (CUD) remains a major public health concern, with well-established acute neuropsychiatric, cardiovascular, and systemic effects [1]. Cocaine, a potent psychostimulant, primarily acts by blocking the dopamine (DAT), serotonin (SERT), and norepinephrine (NET) transporters, thereby rapidly increasing synaptic monoamine levels [2]. Although the immediate effects of euphoria, psychomotor activation, and craving are well characterised, scientific focus has increasingly shifted towards the long-term neurobiological consequences of sustained exposure [3].

Emerging evidence suggests that chronic cocaine use may be associated with progressive cerebral changes that partly resemble neurodegenerative mechanisms [4,5,6,7,8,9]. Structural and functional neuroimaging studies reveal deviations from typical trajectories observed in neurodegenerative disorders such as Alzheimer’s disease (AD) and Parkinson’s disease (PD) [10,11,12]. Although these links remain preliminary, they support the idea that cocaine-related brain injury may involve mechanisms distinct from—rather than simply accelerating—conventional neurodegenerative processes.

Within this interpretative framework, the concept of a “cocaine-related cerebropathy” or “cocaine-specific neurodegeneration” is presented as a provisional and heuristic construct.

The term cocaine-specific cerebropathy is used here solely as a heuristic framework to describe a state of heightened neurobiological vulnerability and does not imply a distinct clinical entity, diagnostic category, or established neurodegenerative process.

This potential condition is characterised by selective involvement of the prefrontal cortex, striatum, limbic structures (including the hippocampus and amygdala), and the cerebellum. It is theorised to arise from converging pathways, including dopaminergic dysregulation, chronic neuroinflammation, oxidative stress, mitochondrial impairment, glutamatergic excitotoxicity, and blood–brain barrier disruption [8,13]. The construct is conceptual rather than diagnostic, intended to encourage empirical refinement of classifications of substance-related brain disorders.

Individual vulnerability is a key factor in the onset and progression of cocaine-related brain changes. Neurodevelopmental conditions, particularly attention-deficit/hyperactivity disorder (ADHD), increase susceptibility through delayed cortical development, impaired prefrontal integration, and reduced neurobiological resilience [14,15,16]. The “Last-In, First-Out” (LIFO) neurodevelopmental model offers a helpful perspective: associative and prefrontal networks, which mature late, may be among the first to decline under chronic neurotoxic, vascular, or inflammatory stressors [17,18,19]. The link between ADHD and substance use disorders is well established [20,21], and recent hypotheses propose that individuals with ADHD may not only be more prone to cocaine use but also more vulnerable to its potential neurodegenerative effects. Applying the LIFO framework to cocaine-related brain damage could help identify high-risk groups and inform preventative strategies.

Importantly, neurodevelopmental vulnerability is only one aspect of a broader risk landscape that includes infectious diseases (notably HIV), genetic predispositions to neurodegenerative trajectories, vascular and metabolic comorbidities, traumatic brain injury, polysubstance exposure, psychiatric comorbidities, and chaotic life rhythms, including malnutrition and sleep disorders [22,23,24,25,26,27,28,29,30,31]. From this perspective, cocaine-related brain effects may be better understood as the result of a multi-hit interaction between ongoing stimulant use and pre-existing biological vulnerabilities, rather than a single toxic effect.

The aim of this *perspective* is to present an integrated theoretical model of cocaine-related neurodegenerative vulnerability that encompasses both the direct neurobiological effects of prolonged stimulant use and the individual profile of vulnerability that shapes susceptibility, severity, and progression. Rather than viewing chronic cocaine use solely through the lens of addiction, this Perspective reframes it as a potential risk state for neurodegenerative vulnerability—rather than as a primary neurodegenerative disease entity—in which cumulative toxic, vascular, inflammatory, and developmental factors interact with cocaine’s pharmacological effects to weaken neural resilience and, in susceptible individuals, promote degeneration-like trajectories.

By outlining this conceptual shift, the perspective aims to refine the boundaries between substance-induced disorders and neurodegenerative conditions, stimulate hypothesis-driven research, and guide future efforts in early detection, risk stratification, and preventive intervention. The proposed model is explicitly heuristic and exploratory, designed to support empirical testing rather than to assert a new diagnostic entity.

## 2. Methods

This Perspective was developed through a structured, critical, and interpretative synthesis of peer-reviewed literature examining the neurobiological consequences of chronic cocaine exposure and its potential association with neuroprogressive vulnerability. Targeted searches were conducted in PubMed, Scopus, and Web of Science from database inception to December 2025. Search strategies combined keywords and controlled vocabulary terms related to cocaine and cocaine use disorder, chronic or repeated exposure, neuroimaging (MRI, DTI, fMRI, PET, SPECT), neuropathology and post-mortem findings, neuroinflammation, oxidative stress, mitochondrial dysfunction, blood–brain barrier integrity, cognitive and motor outcomes, and neurodegenerative disorders (including Alzheimer’s disease, Parkinson’s disease, dementia with Lewy bodies, and frontotemporal dementia). Reference lists of key articles and relevant reviews were manually screened using a snowballing approach to ensure comprehensive coverage of mechanistically relevant studies. To enhance transparency and address variability in the literature, the major evidence domains, degree of empirical support, and principal sources of inconsistency are summarised in Supplementary Table S1.

Given the hypothesis-generating and conceptual nature of this Perspective, inclusion criteria were intentionally broad. Studies were considered eligible if they examined repeated or chronic cocaine exposure or cocaine use disorder in humans or experimental models, reported outcomes relevant to brain structure, connectivity, neurochemistry, neuroinflammation, oxidative or mitochondrial mechanisms, neuropathology, cognitive performance, or motor function, and provided mechanistic or systems-level relevance to fronto–striato–limbic and/or cerebellar networks or to biological pathways implicated in neurodegenerative vulnerability. Exclusion criteria included studies limited exclusively to acute intoxication without relevance to chronic brain outcomes, reports lacking neurobiological, neurological, or cognitive endpoints, and studies in which cocaine exposure could not be meaningfully distinguished from other primary etiologies when this precluded interpretation. Non-peer-reviewed sources were not considered. Case reports and small clinical series were included selectively when they provided clinically or mechanistically informative observations relevant to the proposed framework.

As this work is a conceptual Perspective rather than a systematic review or meta-analysis, no formal quantitative risk-of-bias instrument was applied. Instead, evidence was appraised qualitatively by assigning greater interpretative weight to longitudinal studies, meta-analyses, and multimodal investigations when available, and by prioritising convergent findings across independent methodologies, such as human imaging studies combined with preclinical mechanistic data. Key confounders were explicitly considered throughout the synthesis, including polysubstance use, psychiatric and medical comorbidities (notably HIV infection), cardiometabolic burden, age, duration of abstinence, and socioeconomic factors. Particular attention was given to evidence regarding reversibility with sustained abstinence, as this represents a central point of divergence in the literature.

Conflicting or null findings were not excluded; instead, discrepancies were examined in relation to sample characteristics and vulnerability profiles, definitions and quantification of cocaine exposure (dose, duration, and route of administration), imaging and analytical methodologies, control of confounding variables, and duration of abstinence at the time of assessment. Where studies suggested partial structural or functional recovery, these findings were explicitly integrated into the interpretation of cocaine-related neuroprogression as a conditional and non-mandatory trajectory, consistent with a vulnerability-based model rather than a deterministic degenerative pathway. This synthesis does not introduce new empirical data but integrates existing findings into a heuristic framework intended to guide future hypothesis-driven research. The proposed model of cocaine-related neurodegenerative vulnerability is explicitly exploratory and should not be interpreted as establishing a distinct diagnostic entity.

## 3. Results

### 3.1. Neurochemical Mechanisms of Cocaine Action and Modulators of Individual Vulnerability

Cocaine acts through a complex interplay of neurochemical, vascular, metabolic, and inflammatory mechanisms [2]. These acute and chronic actions interact with pre-existing biological vulnerabilities, resulting in diverse trajectories of brain dysfunction and potential neurodegeneration. Individual susceptibility is shaped by genetic predispositions, neurodevelopmental differences, age, cardiometabolic and cerebrovascular risks, immune status (including HIV infection), traumatic brain injury, and polysubstance exposure.

Cocaine is a potent, non-selective inhibitor of the dopamine (DAT), norepinephrine (NET), and serotonin (SERT) transporters, leading to a rapid build-up of extracellular monoamines within fronto–striato–limbic circuits. Emerging evidence also suggests this may occur within cerebellar pathways [32,33,34,35]. These acute disturbances underpin cocaine’s reinforcing properties and create conditions that could destabilise circuits, potentially resulting in neurotoxic processes in vulnerable individuals.

Cocaine’s primary dopaminergic effects arise from DAT inhibition, resulting in prolonged hyperstimulation across mesolimbic, mesocortical, and nigrostriatal pathways [36,37,38,39]. Although direct dopaminergic projections to the cerebellum are relatively sparse, preclinical studies indicate potential dopaminergic modulation of cerebellar plasticity and motor–cognitive integration [32]. These findings are preliminary, but they suggest that dopaminergic stress may extend beyond traditionally implicated networks.

In serotonergic and noradrenergic systems, cocaine increases monoaminergic activity by blocking SERT and NET [40,41,42,43,44]. Alterations in 5-HT2A/5-HT2C signalling contribute to hyperlocomotion, compulsive drug seeking, and autonomic dysregulation, while increased noradrenergic activity heightens arousal and cardiovascular stress. Although less well understood, serotonergic and noradrenergic projections to the cerebellum suggest that monoaminergic effects may extend beyond cortical and limbic regions, particularly in individuals with existing vulnerabilities [45,46,47].

Cocaine also interacts with Sigma-1 receptors (Sig-1R), which regulate calcium signalling, synaptic plasticity, and neuroimmune responses [48,49,50,51]. Sig-1R expression is observed in both cortical and subcortical regions, and in some studies within cerebellar tissue [52]. Although the functional importance of cerebellar Sig-1R activation remains unclear, its potential role is consistent with broader sigma-mediated stress pathways.

Cocaine further disrupts glutamate homeostasis by altering NMDA receptor subunits and impairing glutamate clearance [53,54]. This leads to hyperexcitability, increasing susceptibility to excitotoxic injury—a pathway implicated in several neurodegenerative disorders. The cerebellum, which relies heavily on glutamatergic transmission, may also be at risk, although direct human evidence remains limited.

Furthermore, cocaine affects the endogenous opioid system by activating μ- and κ-opioid receptors, which modulate reward, stress responses, and dysphoria during withdrawal [55,56,57]. The presence of opioid receptors within cerebellar networks suggests potential, yet not fully examined, effects on both emotional and motor control.

Beyond its effects on neurotransmission, cocaine blocks voltage-gated sodium channels, increasing the risk of seizures and altering neuronal excitability.

At the vascular level, sodium and calcium channel blockade, together with potent vasoconstriction, increases the risk of ischaemic and cerebrovascular events, with secondary effects on metabolically vulnerable brain regions [58].

These neurochemical mechanisms do not operate in isolation but intersect with vulnerability factors that shape both acute responses and long-term trajectories. Neurodevelopmental disorders such as ADHD or ASD may delay cortical maturation and reduce neural resilience; genetic variants can amplify dopaminergic or glutamatergic toxicity; cardiometabolic and immunological comorbidities increase oxidative and vascular burdens; traumatic brain injury and polysubstance use further diminish compensatory capacity. Although less well characterised, similar dynamics may affect cerebellar vulnerability, particularly in individuals with pre-existing neurodevelopmental or vascular fragility.

Taken together, the mechanisms described outline a broad and interacting set of neurochemical, vascular, metabolic, and inflammatory pathways through which cocaine may exert acute and long-term effects on brain function. Our interpretative appraisal underscores that the evidence for dopaminergic, serotonergic, noradrenergic, and glutamatergic contributions to cocaine-related neurotoxicity is comparatively strong. In contrast, proposed roles for cerebellar monoaminergic modulation, Sigma-1 receptor signalling, and cerebellar opioid mechanisms remain preliminary, derived largely from early-stage or preclinical investigations [59,60,61,62,63,64].

Similarly, while vulnerability factors—including neurodevelopmental conditions (not only neurodevelopmental conditions but also other psychiatric vulnerabilities, including psychotrauma), cardiometabolic burden, HIV infection, traumatic brain injury, and polysubstance exposure—offer a plausible framework for differential susceptibility, the causal pathways linking these factors to specific neuroprogressive or degenerative outcomes remain insufficiently defined. Although excitotoxicity, oxidative stress, and neuroinflammation are well-established consequences of stimulant exposure, their capacity to induce sustained or degenerative changes in humans has yet to be conclusively demonstrated.

Overall, the current synthesis supports a neuroprogressive risk hypothesis rather than a confirmed neurodegenerative trajectory, underscoring the need for longitudinal, multimodal research before establishing a comprehensive model of cocaine-related neurodegeneration.

**Figure 1:**
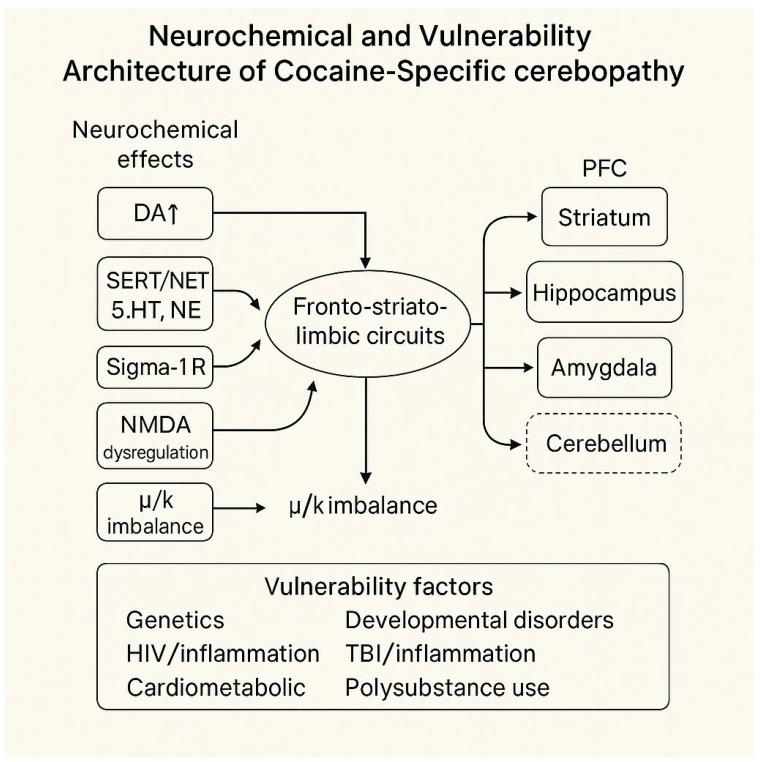
Neurochemical and vulnerability architecture underlying cocaine-specific cerebropathy. Arrows indicate functional interactions and influence among the neurochemical systems and vulnerability factors involved in the development and progression of cocaine-related cerebropathy. DA = Dopamine; SERT = Serotonin Transporter; NET = Norepinephrine Transporter; 5-HT = 5-Hydroxytryptamine (Serotonin); NE = Norepinephrine; Sigma-1R = Sigma-1 Receptor; NMDA = N-Methyl-D-Aspartate Receptor; μ/k = Mu/Kappa Opioid Receptors; PFC = Prefrontal Cortex; HIV = Human Immunodeficiency Virus; TBI = Traumatic Brain Injury.

The overall organisation of these interacting mechanisms is shown in Figure 1.

The main neurotransmitter systems involved in cocaine exposure, along with their associated vulnerability profiles, are summarised in Table 1.

### 3.2. Neuropathophysiological Mechanisms Underlying Cocaine-Specific Cerebropath

Chronic cocaine exposure appears to trigger a range of neurochemical, vascular, metabolic, and inflammatory mechanisms that may act as causal, accelerating, or anticipatory factors in neurodegenerative-like processes. These mechanisms could give rise to diverse clinical phenotypes, shaped by patterns and duration of use as well as the individual’s baseline neurobiological resilience. Vulnerability to cocaine-related brain injury is thus unlikely to be uniform; instead, it seems to reflect a complex interplay between long-term stimulant exposure and pre-existing biological vulnerabilities, including genetic predispositions, age-related neural fragility, cardiometabolic and vascular risks, infectious conditions such as HIV, traumatic brain injury, neurodevelopmental disorders, and polysubstance use. Each of these factors may lower the threshold for neuronal dysfunction, and, when combined with cocaine’s neurotoxic effects, could hasten neurodegenerative trajectories.

Within this tentative conceptual framework, a cocaine-specific cerebropathy can be described as arising through two main interconnected pathophysiological routes. The first involves fronto–striato–limbic dopaminergic imbalance, in which sudden monoaminergic increases are followed by presynaptic and postsynaptic neuroadaptations that may gradually impair dopamine signalling, synaptic stability, and neuronal health. The second involves a broader neurotoxic cascade linked to oxidative stress, mitochondrial dysfunction, excitotoxicity, neuroinflammation, impaired vesicular monoamine storage, and vascular issues, which may lead to multifocal structural and functional changes.

These proposed pathways appear to converge on neural regions characterised by high metabolic demand, dense synaptic architecture, or prolonged developmental timelines. Late-maturing structures—including the prefrontal cortex, associative cortices, striatal loops, limbic regions, and possibly cerebellar networks—may therefore be particularly vulnerable. This vulnerability may be further heightened in individuals with neurodevelopmental conditions such as ADHD, where delayed cortical maturation, atypical synaptic pruning, and fronto-striatal dysconnectivity might reduce compensatory capacity and increase susceptibility to neurotoxic, inflammatory, or vascular insults.

Taken together, the interaction between chronic cocaine use and antecedent vulnerability factors suggests a multiple-hit model in which neurochemical dysregulation and limited neural resilience may jointly increase susceptibility to neurodegenerative-like outcomes. These dynamics provide a potential framework for conceptualising the pattern of cerebral involvement tentatively described as cocaine-specific cerebropathy.

#### 3.2.1. Dopaminergic System Alterations

Among the neurochemical systems potentially affected by cocaine, alterations in dopaminergic signalling are often regarded as a key factor linking immediate reinforcement processes with long-term neuroadaptations and, in some cases, suspected neurodegenerative pathways. Cocaine primarily targets dopamine-rich circuits connecting the ventral tegmental area, nucleus accumbens, prefrontal cortex, and dorsal striatum, producing rapid increases in extracellular dopamine, followed by a series of compensatory adjustments. In this context, it is important to distinguish between immediate responses driven by dopamine transporter (DAT) blockade and long-term adaptations involving presynaptic, postsynaptic, mitochondrial, and structural changes when considering the hypothesis of cocaine-specific cerebropathy [65,66].

Cocaine acutely inhibits DAT, causing rapid increases in extracellular dopamine in striatal and cortical regions [67]. This elevation enhances activation of D1- and D2-like receptors and is widely regarded as central to cocaine’s euphoric and reinforcing properties [68,69]. Blockade of SERT and NET further boosts serotonergic and noradrenergic tone, influencing mood, arousal, autonomic responses, and the significance attributed to drug-related cues [70,71,72]. Preclinical findings also suggest that cocaine might modulate dopamine receptor signalling, including possible allosteric enhancement of D2 receptor activity, although these mechanisms are less clearly understood in humans [73]. The resulting transient hyperdopaminergic state is characterised by increased reward sensitivity and psychomotor activation. Individuals with genetic variants, neurodevelopmental traits, or prior stimulant exposure may experience greater destabilisation under these conditions [74,75]. These initial responses might represent an early step in a broader series of dopaminergic changes.

Repeated cocaine exposure appears to induce more persistent neuroadaptations in fronto–striato–limbic circuits. Increased striatal DAT binding observed in post-mortem and imaging studies is considered a compensatory response to recurrent transporter blockade [76,77]. Reduced D2/D3 receptor availability, consistently reported in PET studies of individuals with cocaine dependence, has been linked to impulsivity, compulsive drug seeking, and decreased responsiveness to natural rewards [78,79,80]. At the intracellular level, chronic cocaine exposure has been associated with mitochondrial dysfunction, oxidative stress, and heightened vulnerability to metabolic injury [81,82,83]. Additional transcriptional and structural changes affecting dendritic spine density and synaptic organisation within medium spiny neurons have also been documented [84,85,86,87]. Overall, these findings indicate a shift towards a dysregulated, often hypodopaminergic state that may increase susceptibility to neurotoxic processes in vulnerable individuals.

Presynaptic adaptations include evidence of DAT up-regulation, with post-mortem and animal studies reporting enhanced dopamine uptake capacity after chronic exposure [88,89,90,91]. Increased α-synuclein expression has also been observed in midbrain dopaminergic neurons and striatal synaptosomes [92,93,94,95,96], raising the possibility that cocaine may influence protein systems involved in dopamine regulation. Experimental studies suggest that α-synuclein may modulate DAT trafficking and intracellular dopamine handling, potentially fostering oxidative stress and reducing neuronal resilience [97,98,99]. Findings from synucleinopathy models further indicate that increased α-synuclein burden can disrupt firing properties, calcium dynamics, dopamine release, and neuronal morphology [100,101], supporting cautious consideration of α-synuclein as a possible contributor to neurodegenerative vulnerability in the context of chronic stimulant exposure [97,102].

Postsynaptic adaptations have also been observed, particularly decreases in D2/D3 receptor availability that persist during abstinence and may reflect attempts to reduce excessive dopaminergic stimulation [78,79,103,104,105,106]. These changes have been linked to anhedonia, impulsivity, compulsive drug seeking, and reduced cognitive control [80,107,108]. Early imaging studies suggest that similar adaptations might occur outside traditional dopaminergic networks, with reports of cerebellar structural and connectivity alterations in chronic users [32,33,109,110,111,112], although the evidence remains preliminary.

Structural imaging studies have also reported putaminal hypertrophy in some individuals with chronic cocaine use [113,114,115]. This enlargement has been interpreted as reflecting compensatory or maladaptive plasticity within basal ganglia circuits. Further investigations have identified region-specific striatal increases alongside cortical and cerebellar reductions [116,117,118]. These changes may reflect dendritic arborisation, synaptic reorganisation, or glial responses, although their functional significance remains uncertain. Some studies have linked putaminal alterations to dyskinesias, stereotyped behaviours, and impaired motor or cognitive control [119,120].

Taken together, these mechanisms provide a broad but necessarily provisional explanation of how dopaminergic changes might develop during both acute and chronic cocaine use. While the synthesis offers a clear, hypothesis-driven framework that incorporates presynaptic, postsynaptic, mitochondrial, and structural findings, the supporting evidence remains inconsistent. Much of the data on α-synuclein dynamics, mitochondrial dysfunction, cerebellar involvement, and putaminal hypertrophy come from preclinical or cross-sectional studies, limiting the ability to establish causal or progressive relationships. Even well-established findings, such as reductions in D2/D3 receptor availability, cannot yet be assumed to indicate neurodegeneration rather than reversible or fluctuating neuroadaptation. Therefore, applying cellular and animal observations to human pathology requires ongoing caution, especially when considering multi-hit models that combine acute dopaminergic surges, long-term adaptations, and individual vulnerability factors. For these reasons, the current synthesis should be regarded as an interpretative framework rather than a definitive account of dopaminergic pathology in chronic cocaine use.

Regarding the social aspect of cocaine addiction, social dominance hierarchy position influences brain dopamine D2 receptors and the reinforcing effects of cocaine. Experimental evidence from rodent and non-human primate models indicates that social hierarchy is closely associated with dopaminergic regulation and with differential vulnerability to psychopathology and substance-related behaviours. In isogenic mouse models, individual sociability predicted subsequent social rank, suggesting that pre-existing behavioural traits contribute to the emergence of social organisation. Once hierarchy was established, higher-ranked animals displayed a behavioural profile characterised by increased anxiety, enhanced working memory performance, reduced dopaminergic activity within the ventral tegmental area, diminished behavioural responsiveness to cocaine, and reduced susceptibility to depressive-like phenotypes following repeated social stress. Both pharmacogenetic inhibition of midbrain dopaminergic neurons and genetic disruption of glucocorticoid receptor signalling in dopamine-sensitive brain regions facilitated access to higher social ranks, indicating that the interaction between dopaminergic tone and stress-related neuroendocrine mechanisms plays a central role in shaping social structure and associated behavioural outcomes. Comparable findings have been observed in primate studies, where social status has been shown to modulate dopamine D2/D3 receptor availability and behavioural sensitivity to cocaine. Reorganisation of social groups in cynomolgus monkeys resulted in increased D2/D3 receptor availability in previously subordinate animals who attained dominant status, suggesting that the social environment exerts a plastic influence on dopaminergic function consistent with mechanisms of environmental enrichment. Although overall cocaine self-administration rates did not directly follow social rank after reorganisation, the reinforcing potency of cocaine was reduced in most subjects, indicating a shift in reward sensitivity. Further PET imaging studies demonstrated that social housing selectively increased D2 receptor availability in dominant monkeys and reduced cocaine reinforcement compared with subordinate animals, supporting the notion that environmental and social factors can induce neurobiological adaptations that influence addiction vulnerability [121,122,123,124].

Taken together, these findings support a model in which social environment and hierarchical position interact with dopaminergic signalling and stress-related pathways to modulate behavioural strategies, stress resilience, and susceptibility to substance use disorders. Social status thus emerges not merely as a behavioural outcome but as a biologically embedded condition capable of shaping reward processing and psychopathological risk.

Current experimental evidence indicates that the reinforcing properties of psychostimulants cannot be fully explained by dopaminergic mechanisms alone, although enhancement of dopamine neurotransmission within mesolimbic structures—particularly the nucleus accumbens—remains central. Cocaine and amphetamine-like substances increase extracellular dopamine primarily through dopamine transporter-mediated mechanisms, including reverse transport and inhibition of reuptake. However, converging data suggest that additional non–dopamine transporter-mediated processes substantially contribute to both behavioural activation and reward-related effects. In particular, psychostimulant-induced increases in noradrenergic transmission within the prefrontal cortex appear capable of modifying the firing patterns of midbrain dopaminergic neurons, thereby altering action potential-dependent dopamine release. These changes influence the temporal dynamics of dopamine signalling in the nucleus accumbens, with consequent effects on synaptic integration and plasticity, as dopaminergic modulation of synaptic inputs depends critically on the timing of dopamine release relative to afferent activity.

Long-term exposure to drugs of abuse further induces enduring neurochemical adaptations involving interactions between noradrenergic and serotonergic systems. Repeated administration of cocaine, amphetamine, morphine, or alcohol has been shown to disrupt the reciprocal regulatory relationship between these two neuromodulatory systems, producing persistent sensitisation of both noradrenergic and serotonergic neuronal responses. This process appears to depend on alpha1b-adrenergic and 5-HT2A receptor signalling, as pharmacological blockade of these receptors prevents the development of sensitisation. Notably, similar neurochemical changes are not observed after repeated exposure to non-addictive antidepressants or selective dopamine reuptake inhibitors, suggesting that these adaptations are not solely attributable to increased dopaminergic transmission. These findings support the hypothesis that uncoupling between noradrenergic and serotonergic modulation is a shared neurochemical consequence of repeated exposure to addictive substances and may contribute to long-term vulnerability to relapse.

Behavioural sensitisation models in rodents further support this framework. Repeated psychostimulant exposure produces persistent locomotor sensitisation, accompanied by enhanced cortical norepinephrine release and increased serotonergic reactivity, effects that may persist long after drug discontinuation. Loss of reciprocal inhibition between noradrenergic and serotonergic systems is associated with increased dopaminergic responsiveness and heightened behavioural reactivity to subsequent drug exposure. Importantly, similar mechanisms have been proposed to operate under chronic stress, suggesting that the neurochemical reorganisation induced by repeated drug exposure may overlap with stress-related pathways implicated in the development of psychiatric disorders. Overall, these findings support a multidimensional model of addiction in which dopaminergic reinforcement is embedded within a broader network involving noradrenergic and serotonergic regulation, synaptic plasticity, and stress-related neuroadaptations [125,126,127].

#### 3.2.2. Mitochondrial Dysfunction, Oxidative Stress, Neuroinflammation and Neurotoxicity in Cocaine-Specific Cerebropathy

Chronic cocaine use has been linked to a range of alterations that overlap with the pathological mechanisms observed in several major neurodegenerative disorders, particularly through common pathways of mitochondrial dysfunction and oxidative stress. Experimental results in rodent and cell models suggest that cocaine may increase the production of reactive oxygen species (ROS), impair mitochondrial dynamics, and disrupt bioenergetic homeostasis, particularly within the nucleus accumbens and striatum. These changes can enhance neuronal vulnerability and lead to cell death [82,128,129]. Such observations mirror mechanisms central to neurodegenerative diseases such as Parkinson’s disease, Alzheimer’s disease, Huntington’s disease, and amyotrophic lateral sclerosis, where deficiencies in mitochondrial respiration—particularly at complex I—result in excessive ROS formation, redox imbalance, and progressive neuronal degeneration [130,131,132,133].

Within this framework, oxidative stress appears to interact with other processes relevant to cocaine-specific cerebropathy. Elevated ROS levels may increase the vulnerability of dopaminergic neurons by promoting misfolding and impaired clearance of synaptic proteins implicated in neurodegenerative diseases, as observed in synucleinopathies [134,135,136]. Post-mortem studies in individuals with chronic cocaine use have reported significant overexpression of α-synuclein in midbrain dopaminergic regions, a finding that may reflect a maladaptive response to increased dopamine turnover and oxidative stress, thereby affecting proteostasis and neuronal resilience [8,92]. This interaction between oxidative stress and α-synuclein accumulation could impair mitochondrial function and protein clearance pathways, creating conditions conducive to degenerative changes within dopaminergic circuits.

Another area of interest is the vesicular monoamine transporter 2 (VMAT2), which is vital for sequestering dopamine into synaptic vesicles and reducing its cytosolic oxidation. Reduced VMAT2 function raises cytosolic dopamine levels, thereby increasing ROS formation and mitochondrial damage. In genetic and toxin-based models, decreased VMAT2 expression has been shown to cause progressive nigrostriatal degeneration and Parkinsonian phenotypes, preceding detectable changes in DAT or D2 receptor binding [137,138,139,140,141]. Although direct in vivo evidence for VMAT2 in cocaine use disorder remains limited, the combination of high dopamine turnover, oxidative stress, and possible VMAT2 dysregulation offers a plausible mechanistic link between stimulant exposure and dopaminergic vulnerability.

Mitochondrial dysfunction and oxidative stress may also contribute to broader neuroinflammatory responses. Preclinical and translational studies show that cocaine can activate microglia in dopamine-rich regions—including the nucleus accumbens, prefrontal cortex, and hippocampus—while increasing expression of pro-inflammatory mediators such as HMGB1–RAGE and NF-κB-dependent cytokines [129,142,143,144,145]. Cocaine-induced oxidative stress and inflammation may further weaken the blood–brain barrier (BBB), allowing peripheral immune cell entry and enhancing central toxicity. Reviews of substance use disorders have highlighted chronic psychostimulant exposure as a significant factor in BBB disruption and neurovascular dysfunction [146,147,148]. Some experimental studies suggest that antioxidant or anti-inflammatory interventions—such as N-acetylcysteine—can reduce cocaine-related mitochondrial damage and microglial activation, indicating that this pathway may be therapeutically modifiable [149].

Taken together, these observations support the hypothesis that mitochondrial impairment, oxidative stress, and neuroinflammation may constitute a second major pathophysiological pathway relevant to cocaine-specific cerebropathy. In individuals with pre-existing genetic susceptibilities, cardiometabolic or infectious comorbidities, or neurodevelopmental fragility, these processes may accelerate neurodegenerative trajectories, producing structural and functional changes that could resemble, anticipate, or interact with those observed in primary neurodegenerative disorders. Understanding these shared mechanisms could help identify targets for neuroprotective strategies to reduce the long-term cerebral effects of chronic cocaine use.

While the synthesis above presents a coherent and biologically plausible framework linking chronic cocaine exposure to mitochondrial dysfunction, oxidative stress, and neuroinflammation, much of the supporting evidence derives from preclinical models or post-mortem studies, limiting the ability to infer causality or progression in humans. Although parallels with established neurodegenerative disorders are scientifically suggestive, these similarities do not yet establish a direct mechanistic link between cocaine exposure and neurodegeneration. Findings on α-synuclein accumulation, VMAT2 dysfunction, and BBB disruption, although intriguing, remain inconsistent across studies and often rely on indirect markers rather than longitudinal evidence. Furthermore, the extent to which these changes reflect transient neuroadaptive responses, reversible pathology, or early signs of a degenerative process remains uncertain. Extrapolating from rodent and cellular models to the clinical course of cocaine use disorder should be done cautiously, especially given the heterogeneity of human populations and the influence of polysubstance use, comorbidities, and genetic differences. Nevertheless, the convergence of oxidative, mitochondrial, and inflammatory mechanisms offers a valuable conceptual framework for future research, underscoring areas where rigorous longitudinal and mechanistic studies are essential to clarify their roles in the proposed neuroevolutionary progression associated with chronic cocaine exposure [150].

#### 3.2.3. Structural Plasticity: Spinogenesis and Impaired Neurogenesis

Chronic cocaine exposure has been linked to significant and lasting structural plasticity within striatal circuits, most notably an increase in dendritic spine density on medium spiny neurons (MSNs) in the nucleus accumbens, widely regarded as a hallmark of stimulant-induced neuroadaptation. Animal studies consistently show that repeated cocaine administration promotes the formation and stabilisation of excitatory synapses on MSNs, thereby reshaping the architecture of fronto–striatal reward pathways [84,151,152]. This structural remodelling is not uniform across MSN subtypes; although observed in both D1- and D2-receptor–expressing neurons, it persists particularly in D1-MSNs, which form the direct pathway and are central to reward learning and motivated behaviour. Such preferential involvement may contribute to the imbalance between basal ganglia pathways seen in chronic users, potentially fostering compulsive drug seeking, increased cue reactivity, and impaired inhibitory control [151,153].

At the molecular level, chronic cocaine exposure has been shown to influence transcriptional programmes that regulate synaptic growth. A key mechanism involves suppression of the transcription factor MEF-2, which regulates activity-dependent synaptic pruning. Cocaine inhibits MEF-2 via D1 receptor–mediated signalling, thereby disinhibiting dendritic spine formation. Restoring MEF-2 activity can block these structural changes, suggesting an actively regulated, transcription-driven form of plasticity rather than a passive compensatory process [84]. In parallel, chronic exposure increases ΔFosB, a transcription factor that accumulates selectively in D1-MSNs with repeated drug use and promotes synaptic strengthening and structural reorganisation, potentially reinforcing long-term vulnerability to relapse [84,86,154]. Functionally, these adaptations may amplify glutamatergic drive onto MSNs, destabilise fronto–striatal homeostasis, and bias learning towards drug-related cues, contributing to craving, compulsive intake, and impaired decision-making even after prolonged abstinence [155].

Compared with spinogenesis, the evidence for altered adult neurogenesis in chronic cocaine use remains mixed. Some preclinical studies suggest reductions in the proliferation and survival of neural progenitors in the hippocampal dentate gyrus, changes that may relate to cognitive rigidity, mood dysregulation, or heightened stress responses [156,157]. However, findings vary widely across species, dosing regimens, withdrawal periods, and methodological approaches. Several studies report transient rather than lasting reductions; others fail to find significant effects, and some even describe context-dependent increases [158]. A plausible yet still hypothetical proposal is that cocaine-induced spinogenesis may alter the microenvironment of neurogenic niches; increased synaptic density and altered glutamatergic signalling within the nucleus accumbens have been suggested to influence trophic signals relevant to hippocampal progenitor survival, although existing evidence remains limited and mainly derived from animal models [159]. Currently, the most cautious interpretation is that cocaine may affect neurogenic processes under specific conditions—such as high-dose, long-term exposure or concurrent stress—but confirmed suppression of neurogenesis in humans has yet to be demonstrated.

Collectively, cocaine-induced spinogenesis, particularly in D1-MSNs, is among the most consistent markers of pathological plasticity associated with stimulant exposure, linking molecular transcriptional changes to structural remodelling and lasting behavioural vulnerability. Altered neurogenesis may also contribute to cognitive and emotional dysregulation in some individuals, though current evidence remains inconsistent. Together, these processes demonstrate how chronic cocaine exposure can shift plasticity from adaptive to maladaptive modes, reorganising synaptic networks that may interact with dopaminergic, inflammatory, vascular, and mitochondrial mechanisms within the broader concept of cocaine-specific cerebropathy. The main mechanisms described in this section are summarised in Table 2.

The evidence outlined above presents a coherent and biologically plausible account of cocaine-induced structural plasticity, particularly dendritic spine proliferation in D1-MSNs. However, much of the supporting research derives from animal models, and it remains unclear how well these findings translate to human cocaine users. Although spinogenesis is among the most consistently observed phenomena in preclinical studies, its functional significance in humans—particularly regarding long-term behaviour, relapse risk, and potential neurodegenerative processes—has yet to be clearly defined. Mechanisms involving MEF-2 suppression and ΔFosB accumulation, although compelling, are based on rodent data and should be interpreted with caution when applied to clinical populations. Conversely, evidence for altered neurogenesis is notably diverse and often conflicting, owing to methodological differences and a lack of longitudinal human studies. The idea that synaptic remodelling in the nucleus accumbens could influence neurogenic niches remains speculative and not fully validated. Overall, while structural plasticity is an important aspect of stimulant-related neuroadaptation, current data do not allow definitive conclusions about its role within a broader neurodegenerative process. The mechanisms described should therefore be regarded as provisional within an emerging conceptual framework, rather than as confirmed indicators of cocaine-specific pathology.

#### 3.2.4. Morphological and Functional Brain Changes Associated with Chronic Cocaine Use

Chronic cocaine use has been linked to a cascade of macro- and microstructural brain changes that appear to mirror the complex clinical features of cocaine use disorder and provide tentative support for the concept of a cocaine-specific cerebropathy. Structural MRI studies frequently report reduced grey matter volume in the prefrontal and temporal cortices, hippocampus, amygdala, and striatum, with several analyses noting associations between the severity or duration of use and the extent of these changes [114,116,159,160,161,162,163]. Selective thinning of the superior and middle temporal gyri has been reported, including early work showing reduced cortical thickness in these regions [164] and later morphometric studies indicating dose-dependent reductions [165,166]. These alterations may contribute to deficits in verbal learning, social cognition, and auditory working memory observed in the disorder [167]. Reductions in these regions, often interpreted as reflecting neuronal, synaptic, or dendritic loss, have been associated with impairments in executive functioning, decision-making, and impulse control [116,168]. Large-scale multimodal analyses suggest a pattern of morphometric change that may differ, at least in part, from that seen in other substance use disorders [169,170].

Beyond cortico-limbic regions, converging, though less extensive, evidence suggests that the cerebellum may also be affected by chronic cocaine exposure. Early structural research identified lower cerebellar grey-matter volumes, particularly in the hemispheres, and correlated cerebellar reductions with duration of use and performance on motor or executive tasks [161,171]. More recent studies have replicated and expanded these findings, reporting smaller vermal volumes and preliminary evidence that cerebellar morphology may help distinguish individuals at higher risk of relapse [117,172]. These observations raise the possibility that cerebellar involvement could contribute to disturbances in motor coordination, timing processes, and the regulation of cognitive–affective functions in at least a subgroup of chronic users.

Alterations in white-matter integrity are another consistent finding. Diffusion tensor imaging studies show reduced fractional anisotropy and increased mean diffusivity in major association tracts and interhemispheric fibres—including the corpus callosum, superior longitudinal fasciculus, and frontal white matter—indicating disrupted communication between prefrontal, parietal, limbic, and cerebellar regions [173,174,175]. These abnormalities have been associated with impairments in executive functioning, decision-making, and treatment outcomes, and may partly reflect the combined effects of cocaine and co-exposure to substances such as alcohol or levamisole [176,177,178].

At the systems level, resting-state fMRI studies show altered intrinsic activity and connectivity within major large-scale networks. Dysregulation of the default mode, salience, and central executive networks has been documented, including shifts in the balance between internally and externally directed states and weakened top-down control from prefrontal–cingulate hubs [179,180,181]. Dynamic connectivity analyses further suggest a shift towards DMN-dominant configurations and reduced stability of task-positive states, patterns that correlate with impulsivity, craving, and delay discounting [182,183]. These findings align with structural abnormalities, indicating a sustained reorganisation of functional circuits involved in salience attribution, reward evaluation, and cognitive control [184].

Perfusion and metabolic imaging studies complement this data, indicating that chronic cocaine use may disrupt cerebral blood flow and glucose utilisation beyond the acute vasoconstrictive phase. Hypoperfusion in the prefrontal cortex, anterior cingulate, and hippocampus has been reported, often overlapping with regions of structural loss and associated with deficits in decision-making and affect regulation [185]. FDG-PET investigations—from early studies to recent syntheses—demonstrate reduced glucose metabolism in frontal and cingulate regions during both active use and extended abstinence, supporting the persistence of functional hypofrontality [186,187,188]. Experimental models similarly report region-specific reductions in glucose uptake across cortico-striatocerebellar networks [178,189]. The main structural, connectivity, and metabolic changes are summarised in Table 3.

The structural and functional changes summarised above provide a coherent overview of brain alterations associated with chronic cocaine exposure; however, their interpretation warrants careful consideration. Many findings stem from cross-sectional studies, which limit conclusions about progression over time, causality, or reversibility. Reductions in grey matter in prefrontal and temporal regions are among the most consistent observations, yet it remains unclear whether these reflect neurodegenerative processes, accelerated ageing, neurotoxicity, or pre-existing vulnerabilities. Cerebellar findings—although increasingly confirmed—derive from relatively small cohorts and varied methodologies. White-matter abnormalities identified through DTI likely result from a combination of factors, including cocaine itself, polysubstance use, lifestyle factors, and psychiatric comorbidities, making it difficult to attribute these changes solely to cocaine.

Resting-state network disturbances provide an important systems-level perspective, yet their functional significance remains uncertain because of analytical variability and the influence of state-dependent factors such as withdrawal or recent drug use. Metabolic imaging studies support the possibility of persistent hypofrontality, but it remains unclear whether reduced glucose metabolism reflects a stable trait marker, a reversible neuroadaptive state, or cumulative toxicity. Overall, although the convergence of structural, connectivity, and metabolic abnormalities enhances the plausibility of a cocaine-related cerebropathy, current evidence is insufficient to define a specific or progressive neuropathological entity. Rigorous longitudinal and multimodal research is crucial to elucidate these relationships.

### 3.3. A Proposed Framework for Cocaine-Related Cerebropathy as a Condition of Neurodegenerative Vulnerability

The following advanced-phase description is not intended to imply a unified or inevitable neurodegenerative syndrome, but to explore a hypothetical extreme of cumulative vulnerability observed in a minority of cases.

The existing scientific literature increasingly reports associations between chronic cocaine use and a higher risk of developing motor and cognitive conditions that partially resemble recognised neurodegenerative disorders [167,190,191,192,193]. Although these findings do not establish a causal or consistent pattern, they have led to the hypothesis that long-term stimulant exposure may, in some predisposed individuals, contribute to processes that resemble or interact with neurodegenerative mechanisms affecting specific neural systems.

The proposed framework integrates the neurobiological effects of chronic cocaine exposure with pre-existing vulnerabilities—genetic, neurodevelopmental, vascular, inflammatory, or age-related—highlighting that neuroprogressive risk arises not solely from cocaine but from the interaction between stimulant exposure and baseline fragility.

Regarding the motor aspect, chronic cocaine use has been linked to a range of abnormalities, from parkinsonian signs—such as rest tremor, rigidity, and bradykinesia—to cerebellar dysfunction, including ataxia, dysmetria, and dysarthria, and, in some cases, choreiform or choreoathetoid movements resembling those observed in established movement disorders [191].

On the cognitive–behavioural level, several reports have described an association between long-term cocaine use and an increased risk of conditions such as Alzheimer’s disease, dementia with Lewy bodies—characterised by cognitive fluctuations, visual hallucinations, and parkinsonism—and frontotemporal dementia, which is marked by impairments in executive function, behaviour, and language [194]. These clinical features are highly diverse, and their appearance cannot be solely attributed to cocaine exposure; however, the range of manifestations has led to speculation that, in some individuals, chronic use might contribute to a widespread and potentially progressive pattern of brain dysfunction.

Such dysfunction, if it occurs, likely results from a complex interplay of vulnerability factors, including genetic differences (such as dopaminergic polymorphisms, APOE variants, and synuclein-related variants), neurodevelopmental conditions (such as ADHD or autism spectrum disorders), cardiometabolic and systemic inflammatory states, infectious comorbidities (HIV/HCV), and patterns of cocaine use over time [195,196,197,198,199,200]. The convergence of these pre-existing vulnerabilities with the neurochemical, vascular, inflammatory, and structural effects of cocaine may, in some cases, create a state of heightened neurodegenerative susceptibility—a conceptual framework tentatively referred to here as cocaine-specific cerebropathy [150].

It is crucial to emphasise that this proposal requires careful epistemological consideration. The notion of a relatively defined or progressive clinical trajectory remains speculative, based on converging but not yet fully systematised observations. Available data currently suggest only that clinical evolution might, in a non-mandatory and highly variable manner, follow recognisable patterns, although significant interindividual variation is expected and definitive staging models cannot yet be established.

The following three-phase framework is not intended as a clinical staging system but as a heuristic tool to tentatively organise diverse clinical and biological data.

#### 3.3.1. Early Phase: A Provisional Model of Initial Neuropsychiatric and Neurofunctional Disruption

In the earliest stage of the proposed framework, the clinical presentation is characterised primarily by neuropsychiatric symptoms spanning a wide phenomenological spectrum. Mood symptoms may present as depressive, mixed, or hypomanic episodes, often accompanied by anhedonia, apathy, irritability, and affective lability. Anxiety-related issues are common, ranging from generalised anxiety to panic attacks and agoraphobic traits, and are accompanied by sleep disturbances and significant circadian dysregulation. Features resembling reward-deficiency states may be present, and in vulnerable individuals, transient or persistent psychotic symptoms or obsessive–compulsive phenomena have also been observed [201,202,203,204,205,206,207,208].

As with other central nervous system disorders, cocaine use disorder appears to have a recognisable psychopathological profile, plausibly linked to the stimulant’s effects on neural systems that regulate motivation, salience attribution, emotional processing, and executive functions. Limiting its description to craving, tolerance, and withdrawal risks underestimates the complexity of early neurofunctional dysregulation. Affective, anxious, psychotic, or obsessive–compulsive symptoms may not only be behavioural signs but also early indicators of circuit-level disruption [209,210]. This complexity can lead to diagnostic uncertainty or misinterpretation under the label of dual diagnosis. In some cases, such presentations might instead represent the initial phase of a neuroprogressive pathway, in which genetic, neurodevelopmental, or temperamental vulnerabilities interact with stimulant neurotoxicity, producing a hybrid clinical profile that challenges conventional nosological boundaries.

Despite this heterogeneity, some degree of partial reversibility usually persists in the early stage. Emotional and cognitive symptoms may improve with integrated treatment programmes that combine pharmacological, psychotherapeutic, and psychoeducational interventions, alongside structured treatments for cocaine use disorder, provided sustained abstinence is maintained. Individuals with greater biological vulnerabilities—such as neurodevelopmental conditions, a family history of mood disorders, or cardiometabolic fragility—may show more limited reversibility, emphasising the importance of timely intervention to prevent progression to later stages.

From a neurobiological perspective, this initial phase may reflect early yet significant disruption across several interconnected functional circuits. Early changes occur within reward and motivation systems—particularly the VTA–nucleus accumbens–prefrontal axis—where repeated cocaine exposure can induce a paradoxical functional pattern characterised by hypersensitivity to drug-related cues and reduced responsiveness to natural rewards. This may contribute to anhedonia, apathy, and craving [211,212,213,214,215]. Simultaneously, emerging dysfunction within the salience network—including the insular cortex and anterior cingulate cortex—may heighten negative interoceptive states and reduce attentional flexibility, fostering panic–agoraphobic phenomena and emotional lability [216,217,218].

Dysregulation of the hypothalamic–pituitary–adrenal axis is another potential contributor. Repeated cocaine-induced activation may lead to sustained increases in cortisol and impaired stress regulation, which could underpin emotional fragility, insomnia, dysphoria, and affective instability, and interact with dopaminergic and serotonergic changes [219,220,221,222]. Early neurochemical changes may include dopaminergic hyperactivity, followed by receptor downregulation and tonic hypoactivity, coupled with serotonergic and noradrenergic disruptions that affect mood, anxiety, and stress responsivity [223,224].

These neurochemical changes may intersect with early mechanisms that help explain the emergence of psychotic or obsessive–compulsive symptoms, even in the absence of a formal dual diagnosis. Sensitisation of the mesolimbic dopamine system may increase salience attribution and weaken top-down prefrontal regulation [225,226], while disturbances within cortico–striato–thalamo–cortical loops may contribute to intrusive or repetitive cognitive–behavioural patterns characteristic of obsessive–compulsive psychopathology [227,228,229]. Together, these disturbances may influence stimulus selection, sensory gating, and cognitive filtering, fostering neurobiological conditions that could predispose individuals to psychotic-like or obsessive–compulsive–like phenomena [230,231,232].

Neuroimaging studies suggest that even in early stages of chronic exposure, subtle structural alterations may emerge in regions vulnerable to oxidative stress, excitotoxicity, and dopaminergic dysregulation. These changes appear to follow a gradient across prefrontal, limbic, striatal, and cerebellar systems, mirroring early affective, anxious, executive, and psychotic-like or obsessive–compulsive–like symptoms. Early involvement of the dorsolateral and ventromedial prefrontal cortex has been reported, with cortical thinning and reduced grey matter volume correlating with impulsivity, emotional dysregulation, and diminished top-down control [116,162]. Reductions in hippocampal volume, possibly linked to impaired neurogenesis and early memory issues, have also been observed [165], along with increased amygdala reactivity that contributes to anxiety, irritability, and negative salience attribution [214].

Altered white-matter microstructure in major associative tracts—including the superior longitudinal fasciculus and corpus callosum—may reflect early disruption of large-scale network integration and has been linked to executive and attentional vulnerabilities [173,174]. Subtle abnormalities in the caudate, putamen, and orbitofrontal cortex further suggest early impairment of cortico–striato–thalamo–cortical loops involved in psychotic and obsessive–compulsive phenomena [114,116]. Emerging findings also point to cerebellar involvement, including reduced grey matter volume and altered cerebello-prefrontal connectivity, which may contribute to emotional dysmetria, timing disturbances, and early cognitive disorganisation [161,233].

Taken together, these converging observations tentatively suggest that early-stage cocaine-related brain dysfunction may follow a recognisable pattern, aligning with the emerging clinical phenotype and, in some individuals, potentially foreshadowing subsequent cognitive, behavioural, and neuropsychiatric decline.

Although the early-phase framework provides a useful interpretative structure, several methodological considerations limit the strength of causal inference. Much of the evidence derives from cross-sectional neuroimaging, preclinical models, or clinical samples characterised by polysubstance use, psychiatric comorbidity, and variable durations of abstinence—all factors that make it difficult to attribute effects specifically to cocaine. Structural or functional abnormalities linked to early symptoms may predate cocaine exposure, reflecting pre-existing vulnerabilities rather than early neuroprogressive changes. Additionally, neuroimaging alterations in prefrontal, limbic, striatal, and cerebellar circuits are not unique to stimulant exposure and overlap with patterns seen in mood, anxiety, and other substance use disorders. The idea that these clinical and neurofunctional features represent an initial stage of a broader degenerative vulnerability remains plausible but unproven, particularly in the absence of longitudinal studies capable of demonstrating progression or staging. Therefore, the early-phase model should be viewed as a heuristic framework that requires further empirical validation rather than as a definitive clinical concept.

#### 3.3.2. Intermediate Phase: Emerging Executive, Motor and Cerebellar Dysfunction Within a Neuroprogressive Framework

As cocaine exposure becomes more prolonged, the clinical course may progress to an intermediate phase characterised by a more noticeable and identifiable decline in executive functioning, sensorimotor integration, and behavioural regulation. This stage appears to result from an interaction among pre-existing vulnerabilities, accumulated toxicological effects, and neuroprogressive processes likely initiated in the earlier phase. Executive dysfunction often becomes the dominant feature, with gradual impairments in sustained attention, cognitive flexibility, planning, organisation, judgement, and decision-making. These deficits seem to reflect the progressive weakening of fronto–striato–limbic circuits, with diminished top–down prefrontal control occurring alongside heightened limbic responsivity and reduced capacity for internal and external regulation. Individuals with neurodevelopmental vulnerabilities, especially ADHD, may experience an earlier or more severe transition, as cocaine’s disruptive effects further destabilise already less resilient executive networks, thereby fostering impulsivity, disinhibition, and impaired self-regulation.

In parallel, subtle neurological signs may emerge, including clumsiness, fine motor difficulties, mild incoordination, postural instability, and generalised slowing of movement. With ongoing exposure, these subtle signs can progress to more overt motor problems, such as bradykinesia, low-frequency tremor, intermittent rigidity, dyskinesias, or choreo-athetoid movements, together with early cerebellar signs such as dysmetria, ataxia, and scanning dysarthria. These clinical findings align with reports of decreased cerebellar grey matter volume and impaired fronto–striatal structural connectivity in chronic cocaine users [234,235].

A clinically relevant phenomenon in this phase is heightened sensitivity to extrapyramidal side effects from dopamine D2-blocking agents. Individuals may develop rigidity, tremor, or dystonic reactions at unusually low doses—even after minimal exposure—suggesting reduced functional reserve in the nigrostriatal dopamine system. This hypothesis is supported by imaging evidence of decreased striatal D2/D3 receptor availability and impaired dopaminergic tone in chronic cocaine users [171,190,236]. Overall, these manifestations suggest that this intermediate phase may represent a measurable step in a broader neuroprogressive trajectory, with executive dysfunction, emerging motor abnormalities, cerebellar signs, and hypersensitivity to dopaminergic interference reflecting increasing disruption of fronto–striato–thalamo–cortical networks.

From a neurobiological perspective, this stage appears to consolidate and extend the tentative changes observed earlier. Dopaminergic signalling across fronto–striatal and nigrostriatal pathways may deteriorate further, with imaging studies reporting decreased D2/D3 receptor availability, reduced prefrontal regulatory control, and progressive disinhibition of motor and limbic loops. These changes may reflect a shift from predominantly phasic dopaminergic responses to disorganised tonic states, weakening inhibitory control and contributing to impulsivity, emotional instability, and motor dysregulation.

Cerebellar involvement may also become more prominent. Contemporary models emphasise the cerebellum’s role in predictive coding, timing, and cognitive regulation. Structural and functional studies indicate reduced cerebellar grey matter and disrupted dentato–thalamo–cortical connectivity in chronic cocaine use, which may contribute to postural instability, impaired fine motor coordination, dysmetria, and deficits in temporal and sensory prediction.

Glutamatergic signalling may also become increasingly dysregulated, with reduced efficiency of prefrontal glutamatergic projections and heightened vulnerability to excitotoxicity in striatal and thalamic targets. This dysregulation could promote compulsive motor patterns, repetitive behaviours, and cognitive rigidity, consistent with evidence of altered glutamate homeostasis and corticostriatal connectivity [237]. Persistent dysregulation of the hypothalamic–pituitary–adrenal axis may additionally contribute to emotional volatility, irritability, and impaired impulse control, as repeated cocaine-induced activation maintains elevated cortisol levels and heightened circulatory stress reactivity [238].

Structural neuroimaging provides convergent evidence for these neurofunctional dynamics. Progressive thinning of dorsolateral, ventromedial, and orbitofrontal prefrontal regions, loss of hippocampal volume, increased amygdala dysregulation, and deterioration of white matter integrity within associative tracts—including the superior longitudinal fasciculus and corpus callosum—suggest an evolving disconnection syndrome affecting executive, motor, and interhemispheric integration [239,240]. Microstructural changes in the basal ganglia and pallidal circuitry have been associated with bradykinesia, tremor, rigidity, and choreo–athetoid movements, while additional cerebellar volumetric loss and impaired cerebello–thalamo–cortical coupling may contribute to timing deficits and cognitive disorganisation [234].

Overall, the intermediate phase may reflect a neural system gradually losing coherence and regulatory accuracy. The combined deterioration of dopaminergic, cerebellar, glutamatergic, and stress-regulatory mechanisms indicates an increasingly unstable network structure—one that struggles to sustain cognitive control, emotional balance, and motor coordination, potentially setting the stage for more widespread involvement in later stages.

While the framework introduced above offers a structured interpretation of a potential intermediate stage in cocaine-related neuroprogression, several limitations constrain the strength and specificity of the current evidence. Most findings derive from cross-sectional neuroimaging studies, small clinical samples, or preclinical models, each with notable methodological limitations. Many participants in human studies have polysubstance use, psychiatric comorbidities, or medical conditions that independently affect executive functions, motor systems, and white matter integrity, making it difficult to attribute changes solely to cocaine. Moreover, the structural and functional abnormalities described—such as prefrontal thinning, white matter degradation, basal ganglia alterations, and cerebellar involvement—are not unique to cocaine use and are also observed in mood disorders, ADHD, trauma, and other substance use disorders.

Critically, the proposed trajectory remains hypothetical, as longitudinal investigations have not demonstrated progression from early to intermediate phases or established reversibility, inevitability, or prognostic significance. Interindividual variability is substantial, and only a subset of individuals may follow patterns resembling those described. Accordingly, the intermediate-phase model should be viewed as a heuristic framework intended to stimulate further mechanistic research rather than a formal staging system. Rigorous longitudinal, multimodal, and mechanistically informed studies will be essential before firmer conclusions can be drawn about the nature, distribution, or clinical significance of this proposed intermediate phase.

#### 3.3.3. Advanced Phase: Diffuse Cognitive, Behavioural and Motor Impairment Within a Hypothesised Neuroprogressive Trajectory

With prolonged exposure to cocaine spanning years or decades, some individuals may develop widespread multisystem impairment that appears to reflect extensive neuronal and synaptic dysfunction. In this hypothesised advanced stage, cognitive decline may become widespread, affecting episodic and semantic memory, suggesting involvement of the hippocampal and entorhinal regions. Visuospatial disturbances—such as impaired spatial orientation, depth perception, and constructional skills—may arise from disruptions within parietal and occipito–parietal networks. Language abilities may decline, with difficulties in naming, understanding complex sentences, and, in some cases, changes in prosody and discourse organisation, indicating combined frontal and temporal cortical impairment. Executive dysfunction, reduced cognitive flexibility, working-memory issues, and diminished verbal fluency may coalesce into a broader dysexecutive syndrome. Procedural memory may decline alongside increasing apathy and social withdrawal, leading to a progressive loss of functional autonomy. Psychiatric symptoms—including persistent psychotic features or obsessive–compulsive phenomena—may appear or worsen, reflecting altered salience attribution, disrupted gating mechanisms, and compromised fronto–striato–thalamo–cortical organisation. As in earlier stages, premorbid vulnerability seems to play a key role: individuals with genetic risk factors for neurodegenerative disease (e.g., APOE ε4 or synuclein-related variants), neurodevelopmental conditions, chronic systemic inflammation, or previous traumatic or infectious CNS insults may experience earlier or more severe deterioration.

From a neurobiological perspective, this advanced phase may involve a widespread decline in homeostatic capacity across multiple neurotransmitter and cellular systems. Progressive dopaminergic dysfunction may co-occur with degeneration of mesolimbic and nigrostriatal pathways, reductions in D2/D3 receptor signalling, impaired reward processing, and compromised motor control. Serotonergic imbalance within raphe–basal ganglia–prefrontal pathways could contribute to affective flattening, irritability, and increased vulnerability to psychotic episodes. Chronic oxidative stress, mitochondrial dysfunction, and neuroinflammation—often associated with long-term stimulant use—may induce microglial activation and sustained cytokine release, creating a low-grade inflammatory environment resembling mechanisms observed in Parkinson’s disease, dementia with Lewy bodies, and certain forms of frontotemporal degeneration. In some individuals, prolonged cocaine use may interact with dopaminergic vulnerability to promote α-synuclein misfolding or accumulation within midbrain circuits, potentially leading to parkinsonian or mixed degenerative conditions.

Structurally, advanced cases may show widespread atrophy of the frontal, temporal, and parietal cortices, together with progressive degeneration of the hippocampal and limbic regions, which are linked to memory impairment, emotional dysregulation, and apathy. Subcortical structures—including the caudate, putamen, globus pallidus, and thalamus—may show microstructural deterioration consistent with the gradual disintegration of motor and associative circuits. Reduced integrity and metabolic compromise in the ventral tegmental area and nucleus accumbens may accompany these changes. Cerebellar involvement—with significant loss in the vermis and lateral hemispheres and disrupted cerebellum–thalamus–cortical connectivity—may contribute to dysmetria, ataxia, altered motor timing, and cognitive disorganisation. Diffusion tensor imaging may reveal marked reductions in fractional anisotropy across major white-matter tracts—including the corpus callosum, superior longitudinal fasciculus, and fronto–striatal projections—indicating interhemispheric disconnection and progressive breakdown of executive–motor integration. Ventricular enlargement may be observed at advanced stages, reflecting overall parenchymal reduction.

In summary, this advanced-stage formulation offers a cautious, hypothetical account of how long-term neurofunctional dysregulation in chronic cocaine use might progress to widespread neuronal impairment in some individuals. Although individual variation remains substantial, available descriptions suggest a slow, progressive decline in cognitive, behavioural, and motor functions that could ultimately lead to serious disability and a diminished quality of life. Clarification of diagnosis at this stage usually involves integrated neuropsychological, neurological, and neuroimaging assessments, while treatment is mainly supportive. Therefore, prevention and early detection are essential, and future research must focus on identifying early biomarkers and developing strategies to modify or potentially halt the neuroprogressive processes linked to chronic stimulant use.

The advanced-phase framework outlined above should be approached with caution, as the supporting evidence remains incomplete, indirect, and often drawn from diverse methodological sources. Much of the information comes from case reports, small clinical samples, cross-sectional imaging studies, or extrapolations from preclinical research, rather than long-term studies that can clearly demonstrate neurodegenerative progression linked to chronic cocaine use. Patterns of widespread cortical and subcortical atrophy, extensive white-matter damage, and overall cognitive decline are not unique to cocaine use and are commonly seen in other neuropsychiatric, metabolic, and vascular conditions. Additionally, polysubstance use, medical comorbidities, nutritional issues, and episodes of anoxia or cerebrovascular events make it more difficult to identify cocaine’s specific role in the brain changes observed in later stages.

It remains unclear whether the described impairments represent a coherent progression or occur only in a highly vulnerable minority with pre-existing genetic, developmental, or systemic risk factors. The possibility that some late-stage features are due to accelerated ageing, cumulative lifestyle effects, or indirect consequences of chronic illness cannot be ruled out. Therefore, the concept of an advanced stage should currently be treated as a hypothesis rather than a validated clinical entity. Robust longitudinal, multimodal, and mechanistically grounded research will be crucial before confirming the existence, prevalence, or defining features of this potential late phase of cocaine-related neuroprogression.

#### 3.3.4. Potential Therapeutic Strategies Within the Framework of a Proposed Cocaine-Related Cerebropathy

Managing neurodegenerative vulnerability in individuals with chronic cocaine use is a complex clinical challenge, requiring a coordinated, highly personalised approach. Neuropsychiatric symptoms—such as mood instability, anxiety, sleep disturbances, emerging memory issues, and executive dysfunction—may mimic primary psychiatric disorders, complicating diagnosis. At the same time, cocaine use disorder exhibits its own distinct psychopathology, ranging from depressive and anxious symptoms to panic episodes, psychosis, and significant behavioural dysregulation, often masking early signs of a broader neuroprogressive process. In individuals with reduced neural reserve, multimorbidity, or pre-existing biological vulnerabilities, susceptibility to cocaine-related neurotoxicity may be heightened, emphasising the importance of comprehensive assessment covering psychiatric, neurological, and neuropsychological aspects.

Complete abstinence from cocaine forms the essential basis for all subsequent therapeutic strategies. Achieving abstinence usually involves a combination of psychosocial and pharmacological measures. Individual and group counselling, together with participation in structured support programmes, can enhance insight, reduce relapse risk, and support stress regulation [214,238]. Although no pharmacological agent is formally approved for cocaine use disorder, several compounds have shown partial or context-dependent benefits. Psychostimulants such as methylphenidate or lisdexamfetamine may help stabilise dopaminergic tone in individuals with co-occurring ADHD, while bupropion and ropinirole have demonstrated variable effects on craving. Among glutamatergic and GABAergic interventions, topiramate has been linked to reductions in craving intensity, and N-acetylcysteine may support glutamate homeostasis and help mitigate compulsive use patterns. Evidence suggests that alcohol use disorder is associated with disruption of glutamatergic homeostasis, contributing to relapse vulnerability and impaired impulse control. N-acetylcysteine (NAC), a precursor of glutathione capable of modulating glutamate transmission, has been shown in preclinical models to reduce relapse-like alcohol consumption and improve impulse control without significantly affecting overall alcohol intake or motivation to drink. These findings support the hypothesis that NAC primarily acts on neurobiological mechanisms related to relapse prevention and behavioural regulation rather than on reward processes directly. Given its antioxidant properties and its role in restoring neurochemical balance, NAC has emerged as a potential adjunctive treatment in addiction and other neuropsychiatric conditions characterised by impaired glutamatergic regulation, although further clinical validation remains necessary [241,242].

Pharmacological decisions require particular caution. Long-standing stimulant exposure may be associated with reduced striatal dopaminergic reserve, early motor abnormalities, or heightened sensitivity to dopaminergic interference [171,190]. Antipsychotics with high D2-blocking affinity may therefore exacerbate bradykinesia, rigidity, or executive dysfunction, and their use should be restricted to situations of clear necessity, preferably selecting agents with lower D2 occupancy. SSRIs may likewise require careful titration, as they can worsen anhedonia or disrupt reward processing in individuals with compromised mesocorticolimbic signalling. When affective instability prevails, mood stabilisers such as lamotrigine, valproate, or low-dose lithium may be more suitable.

Management of cognitive, neurological, and behavioural sequelae is increasingly important in the context of emerging neuroprogressive patterns. When present, cognitive decline predominantly affects executive function, attention, processing speed, and working memory rather than a primary amnestic profile. Management should therefore focus on neuropsychological rehabilitation to enhance compensatory strategies, promote neuroplasticity, and preserve functional autonomy. In selected cases with mixed or subcortical-like cognitive features, cholinesterase inhibitors may be considered symptomatically; however, their use should not be interpreted as targeting a primary cholinergic deficit, as evidence for cholinergic degeneration in cocaine-related cognitive impairment remains limited. Motor coordination and balance difficulties—particularly when cerebellar involvement is suspected—may benefit from targeted physiotherapy and occupational therapy.

Lifestyle and medical optimisation are key components of the therapeutic framework. A balanced diet, tailored physical activity, proper sleep hygiene, structured stress-reduction strategies, and careful management of cardiometabolic risk factors may confer vital protective effects on neural systems already experiencing chronic dysregulation.

In summary, managing cocaine-related neuroprogressive vulnerability requires a multidimensional, coordinated, and carefully tailored clinical approach. Early identification of patterns potentially linked to prolonged cocaine use, together with timely intervention, may help reduce functional decline. An integrated strategy that combines psychosocial support, carefully selected pharmacotherapies, neurorehabilitation, and structured education for patients and their families provides the most cohesive framework for maintaining function and quality of life in this vulnerable group. A structured overview of these therapeutic methods is presented in Table 4.

## 4. Discussion

Chronic cocaine use in older adults presents an emerging and clinically significant challenge, characterised by a range of neuropsychiatric, cognitive, and motor disturbances that extend beyond the traditional boundaries of substance use disorders. Growing evidence indicates that prolonged stimulant exposure may interact with age-related changes, pre-existing vulnerabilities, and cumulative toxicological burdens to produce a pattern of neurofunctional decline that, in some individuals, resembles or anticipates features of recognised neurodegenerative conditions [190,191,192]. Within this conceptual framework, the idea of a cocaine-related neuroprogressive vulnerability—tentatively known as cocaine-specific cerebropathy—serves as a heuristic model rather than an ostensive category, providing a way to organise the diverse and partially overlapping findings available to date.

Across the literature, numerous mechanisms have been linked to this potential vulnerability, including oxidative stress, excitotoxicity, mitochondrial dysfunction, neuroinflammation, and dysregulation of dopaminergic, glutamatergic, and stress-response circuits [82,128,214,215,237]. These processes may gradually undermine neural resilience, particularly in the ageing brain, where compensatory capacity is already diminished. Clinically, the resulting phenotype encompasses executive dysfunction, affective instability, reward and salience dysregulation, psychotic-like or obsessive–compulsive symptoms, motor slowing or parkinsonism, cerebellar signs, and, in its most advanced stages, global cognitive impairment and reduced autonomy [116,161,201]. Neuroimaging findings—including cortical thinning, hippocampal atrophy, white matter disconnection, and microstructural abnormalities in striatal and cerebellar pathways—provide partial yet significant support for this interpretation [114,165,172,173].

However, the evidence base remains methodologically diverse and often indirect. Much of the available knowledge derives from cross-sectional studies, small or clinically selected samples, preclinical models, or cohorts with polysubstance use and significant comorbidity, making it difficult to isolate cocaine-specific effects [176,177]. Furthermore, several structural and functional changes attributed to chronic stimulant exposure overlap with patterns observed in psychiatric disorders, vascular diseases, or normal ageing. Only a minority of individuals appear to show a clearly progressive pattern, and the boundary between reversible neuroadaptation and irreversible neuronal damage remains poorly understood. Importantly, direct evidence of progressive neuronal loss attributable to chronic cocaine exposure in humans remains limited and largely indirect. While mitochondrial dysfunction and oxidative stress represent the most consistently documented mechanisms overlapping with classical neurodegenerative pathways, other findings—such as dopaminergic dysregulation, α-synuclein alterations, neuroinflammation, and large-scale network changes—are suggestive but insufficient to establish a definitive or self-sustaining neurodegenerative process.

These limitations reflect the uncertainties highlighted in the Critical Appraisal sections: the proposed staging model is conceptually valuable but empirically tentative, and causal conclusions remain premature.

Although the putative mechanisms of cocaine-related neuropsychiatric disorders are numerous, translation into therapeutic strategies remains limited. As described by Venniro et al., experimental models have advanced our understanding of the brain mechanisms of drug self-administration and relapse, yet these mechanistic gains have not translated into improvements in addiction treatment. This problem is not unique to addiction neuroscience, but it is an increasingly common source of disappointment and calls to regroup [243].

From a therapeutic perspective, the framework emphasises early detection, sustained abstinence, and comprehensive multidisciplinary assessment that integrates psychiatric, neurological, and neuropsychological domains. Tailored pharmacological strategies, neurorehabilitation, cognitive remediation, and lifestyle interventions may help stabilise vulnerable circuits and preserve autonomy. Equally essential is identifying individuals with heightened biological susceptibility—whether genetic, neurodevelopmental, vascular, or inflammatory [195,196]—who may require more intensive monitoring and early intervention.

Significant gaps remain. Long-term, multimodal, and mechanistically grounded longitudinal studies are urgently needed to determine whether the alterations described represent transient neuroadaptation, accelerated ageing, or true progressive neurodegenerative trajectories in a subset of vulnerable individuals. Future research should integrate advanced neuroimaging, fluid biomarkers [8], genetic risk profiling, and careful phenotypic stratification to clarify causality, temporal progression, and potential reversibility.

A clearer distinction must be drawn between classical neurodegenerative diseases and the degeneration-like patterns discussed in this framework. Established neurodegenerative disorders are defined by disease-specific proteinopathies (e.g., amyloid-β and tau in Alzheimer’s disease, α-synuclein aggregation in Parkinson’s disease and dementia with Lewy bodies), selective neuronal loss, and predictable neuropathological staging. To date, no consistent evidence demonstrates that chronic cocaine exposure induces such disease-defining proteinopathies or a self-propagating degenerative cascade in humans. Many of the alterations described in this Perspective—particularly dendritic spine remodeling, dopaminergic receptor changes, and large-scale network connectivity shifts—may instead reflect maladaptive neuroplasticity or prolonged neuroadaptive responses rather than irreversible neuronal degeneration.

Ageing-related changes in cognitive reserve, synaptic resilience, and vascular reactivity may represent critical modulators of the proposed vulnerability framework. With advancing age, compensatory capacity within large-scale neural networks progressively declines, reducing tolerance to metabolic stress, inflammatory activation, and dopaminergic imbalance. Individuals with lower cognitive reserve—due to educational, developmental, psychiatric, or vascular factors—may therefore exhibit earlier or more pronounced functional decline under chronic stimulant exposure. Similarly, age-related vascular sensitivity and microvascular fragility may amplify cocaine-induced vasoconstrictive and inflammatory effects, increasing the risk of cumulative network disruption. In this context, cocaine exposure may interact not only with neurodegenerative pathways but also with age-dependent reductions in synaptic resistance and neuroplastic compensation.

Ultimately, recognising late-life cocaine use as a potential neurodegenerative risk condition represents a conceptual development rather than a complete nosological shift. Nonetheless, it encourages clinicians and researchers to adopt a broader perspective on stimulant-related brain changes—one that emphasises vulnerability, prevention, and early intervention as central components of care.

Interestingly, several clinical and experimental aspects associated with cocaine use have also been reported in relation to sleep disorders and deprivation, which are common in CUD (see references below). Therefore, addressing basic needs such as sleep can be helpful in treating CUD and other addictions. However, these important aspects are insufficiently considered in clinical practice, even though all clinicians should be able to manage them. This is all the more important given that there is currently no validated specific medication for CUD [244,245,246,247,248,249,250,251,252,253,254,255].

## 5. Conclusions

Chronic cocaine exposure may increase neurobiological vulnerability rather than produce a primary neurodegenerative disease. Available evidence supports a neuroprogressive risk model in which stimulant-related neuroadaptations interact with ageing, individual susceptibility, and medical or psychiatric comorbidity.

The concept of cocaine-related cerebropathy is therefore proposed as a heuristic framework intended to organise heterogeneous findings without defining a distinct nosological entity. Current data remain insufficient to establish causality or progression, highlighting the need for longitudinal and mechanistically oriented studies.

Early detection, sustained abstinence, and integrated multidisciplinary care remain central clinical priorities in preventing long-term cognitive and functional decline.

## Figures and Tables

**Table 1:** Summary of Neurochemical Systems Implicated in Cocaine Exposure and Relevance to Vulnerability.

Neurochemical System	Primary Mechanisms of Cocaine Action	Neurotoxic Potential	Modulating Vulnerability Factors
Dopamine (DAT → ↑ DA)	Reuptake blockade; D1/D2 overstimulation	Strong (reward, motor, executive circuits)	ADHD, genetic variants of DAT/DRD2, polysubstance use
Serotonin	5-HT2A/2C modulation; salience → compulsivity	Moderate serotoninergic syndrome lower epileptogenic threshold	Mood disorders, ASD traits
Noradrenaline	Hyperarousal; cardiovascular strain	Strong (vasoconstriction, ischemia), brain haemorrhage, lower epileptogenic threshold	Hypertension, cardiometabolic disease
Glutamate	Excitotoxicity; impaired clearance	Strong (structural neurotoxicity) lower epileptogenic threshold	TBI, polysubstance use, inflammation
Sigma-1 receptor	Ca2+ signalling, ER stress, plasticity	Emerging	HIV/inflammation, immune activation
Opioid system (μ/κ)	Reward vs dysphoria imbalance	Moderate	Stress sensitivity, trauma history
Cerebellar circuits	Possible DA/5-HT/GLU modulation	Preliminary	Vascular/metabolic fragility; neurodevelopmental delay

DAT = Dopamine Transporter; DA = Dopamine; D1 = Dopamine D1 Receptor; D2 = Dopamine D2 Receptor; ADHD = Attention-Deficit/Hyperactivity Disorder; DRD2 = Dopamine Receptor D2 Gene; 5-HT = 5-Hydroxytryptamine (Serotonin); ASD = Autism Spectrum Disorder; TBI = Traumatic Brain Injury; ER = Endoplasmic Reticulum; HIV = Human Immunodeficiency Virus; μ = Mu Opioid Receptor; κ = Kappa Opioid Receptor; GLU = Glutamate; ↑ = increase/increased levels.

**Table 2:** Core Neuropathophysiological Mechanisms of Cocaine-Specific Cerebropathy.

Domain	Mechanism	Key Neurobiological Features	Primary Brain Regions	Clinical Implications
1. Dopaminergic Dysregulation	Acute monoaminergic surge	DAT, NET, SERT blockade → ↑ DA/NE/5-HT; phasic dopaminergic overload	Ventral striatum, dorsal striatum, PFC	Euphoria, impulsivity, heightened salience
	Presynaptic adaptations	DAT upregulation; α-synuclein overexpression; impaired DA handling	VTA, SN, striatum	Withdrawal dysphoria, craving, increased neuronal vulnerability
	Postsynaptic adaptations	D2/D3 receptor downregulation; MSN reorganization; basal ganglia imbalance	Dorsal/ventral striatum, PFC	Anhedonia, compulsive use, reduced cognitive control
2. Mitochondrial Dysfunction & Oxidative Stress	ROS overproduction	Complex I impairment; lipid/protein/DNA oxidation	Striatum, NAc, PFC	Neurodegeneration, fatigue, cognitive slowing
	VMAT-2 dysfunction	↓ vesicular DA storage → cytosolic DA toxicity	Striatum	Dopaminergic cell stress, Parkinsonian traits
	Protein misfolding	Oxidative burden → impaired proteostasis, synaptic protein aggregation	SN, VTA	Vulnerability to synucleinopathic mechanisms
3. Neuroinflammation & BBB Injury	Microglial activation	↑ cytokines (TNF-α, IL-1β); oxidative-inflammation loop	PFC, striatum, hippocampus	Cognitive impairment, mood dysregulation
	BBB permeability	Endothelial dysfunction, leukocyte trafficking	Fronto-limbic circuits	Increased neurotoxicity, vulnerability to comorbidities
4. Maladaptive Structural Plasticity	Spinogenesis	↑ dendritic spines (especially D1-MSNs); ΔFosB–MEF-2 imbalance	NAc, dorsal striatum	Cue-reactivity, relapse risk, compulsive behavior
	Altered neurogenesis (inconsistent evidence)	↓ hippocampal progenitor survival (context-dependent)	Dentate gyrus	Memory deficits, emotional instability
5. Cerebellar Vulnerability	Cerebellar-cortical dysconnectivity	Glutamatergic/monoaminergic modulation; Purkinje cell stress	Posterior cerebellum, Crus I–II	Executive dysfunction, timing deficits, motor disorganization
6. Multi-hit Vulnerability Model	Interaction with predispositions	Neurodevelopmental disorders, cardiovascular/metabolic risk, HIV, TBI	Late-maturing circuits	Accelerated neuroprogression; early cognitive decline

DAT = Dopamine Transporter; NET = Norepinephrine Transporter; SERT = Serotonin Transporter; DA = Dopamine; NE = Norepinephrine; 5-HT = 5-Hydroxytryptamine (Serotonin); PFC = Prefrontal Cortex; VTA = Ventral Tegmental Area; SN = Substantia Nigra; D2 = Dopamine D2 Receptor; D3 = Dopamine D3 Receptor; MSN = Medium Spiny Neuron; ROS = Reactive Oxygen Species; NAc = Nucleus Accumbens; VMAT-2 = Vesicular Monoamine Transporter 2; BBB = Blood–Brain Barrier; TNF-α = Tumor Necrosis Factor Alpha; IL-1β = Interleukin 1 Beta; D1 = Dopamine D1 Receptor; HIV = Human Immunodeficiency Virus; TBI = Traumatic Brain Injury; ↑ = increase/increased levels; ↓ = decrease/reduced levels.

**Table 3:** Structural, Connectivity, Network-Level, and Metabolic Alterations Associated with Chronic Cocaine Use.

Domain	Findings	Methods	Clinical Relevance
Grey matter	↓ volume PFC, ACC, hippocampus, amygdala, striatum; thinning temporal cortex	MRI	executive dysfunction, impulsivity, memory impairment
White matter	↓ FA corpus callosum, SLF, frontal WM	DTI	impaired connectivity, poor treatment outcomes
Functional networks	DMN hyperstability; SN/CEN dysregulation	rs-fMRI	impaired cognitive control, craving
Perfusion/metabolism	↓ CBF PFC/ACC; ↓ FDG uptake	SPECT, perfusion MRI, FDG-PET	hypofrontality, anhedonia, decision-making deficits

PFC = Prefrontal Cortex; ACC = Anterior Cingulate Cortex; FA = Fractional Anisotropy; SLF = Superior Longitudinal Fasciculus; WM = White Matter; MRI = Magnetic Resonance Imaging; DTI = Diffusion Tensor Imaging; DMN = Default Mode Network; SN = Salience Network; CEN = Central Executive Network; rs-fMRI = Resting-State Functional Magnetic Resonance Imaging; CBF = Cerebral Blood Flow; FDG = Fluorodeoxyglucose; SPECT = Single Photon Emission Computed Tomography; FDG-PET = Fluorodeoxyglucose Positron Emission Tomography; ↓ = decrease/reduced levels.

**Table 4:** Multidimensional Therapeutic Approaches for Cocaine-Specific Cerebropathy.

Therapeutic Domain	Clinical Targets	Main Interventions
1. Foundational intervention	Abstinence; relapse prevention; stabilisation of addictive behaviour	Integrated psychosocial treatment; motivational interviewing; relapse-prevention strategies; structured peer-support programmes; family involvement and psychoeducation
2. Pharmacological adjuncts in Cocaine Use Disorder	Craving reduction; reward-system dysregulation; executive dysfunction	Psychostimulants in comorbid ADHD; dopaminergic modulators; glutamatergic/GABAergic agents (e.g., topiramate, N-acetylcysteine)
3. Affective and behavioural regulation	Mood instability; dysphoria; anxiety; impulsivity; behavioural dysregulation	Mood stabilisers; cautious antidepressant use; selected antipsychotics with lower D2 affinity; structured behavioural and emotion-regulation interventions
4. Cognitive and neurological symptoms	Memory deficits; executive dysfunction; cognitive slowing; motor symptoms	Cognitive rehabilitation and neuropsychological training; environmental structuring; symptomatic use of cholinesterase inhibitors in selected cases; physical and occupational therapy
5. Sleep and circadian stabilisation	Insomnia; sleep–wake disruption; stress-related arousal	Sleep hygiene programmes; behavioural sleep interventions (CBT-I); circadian-oriented behavioural strategies
6. Lifestyle and long-term care	Neuroprotection; cardiometabolic risk; functional autonomy; quality of life	Physical activity; nutritional optimisation; smoking cessation; cardiometabolic risk management; stress-reduction strategies; multidisciplinary long-term follow-up

Note. Pharmacological interventions in cocaine use disorder are off-label and should be considered adjunctive and individualised within an integrated psychosocial framework. ADHD = Attention-Deficit/Hyperactivity Disorder; GABA = Gamma-Aminobutyric Acid; D2 = Dopamine D2 Receptor; CBT-I = Cognitive Behavioural Therapy for Insomnia.

## Data Availability

No new data were created or analyzed in this study.

## Supplementary Materials

The following supporting information can be downloaded at: https://www.mdpi.com/article/10.3390/jcm15062222/s1, Table S1: Degree of empirical support across domains relevant to the proposed cocaine-related neuroprogressive vulnerability model.
