# Supplementary material for: From Neuroadaptation to Neuroprogression: Rethinking Chronic Cocaine Exposure Through a Model of Cocaine-Related Cerebropathy
Source: J Clin Med. 2026 Mar 14;15(6):2222. doi: 10.3390/jcm15062222 (PMC13026448; doi:10.3390/jcm15062222)
Supplement: Supplementary file 1 [file jcm-15-02222-s001.zip › jcm-4181822-supplementary.pdf]

Table S1. Degree of empirical support across domains relevant to the proposed cocaine-related neuroprogressive vulnerability model

Domain	Main evidence base	Typical direction of findings	Overall level of support*	Key sources of uncertainty / common confounders	Key references (examples)
**Dopaminergic adaptations**(D2/D3; endogenous DA tone)	Human PET + translational imaging	Reduced D2/D3 availability; reduced endogenous DA tone in dependence	**Strong**	Cross-sectional designs; abstinence duration; comorbidities; polysubstance exposure	Martinez et al., 2004; Martinez et al., 2009; Volkow et al., 1990
**Dopamine transporter regulation**	Post-mortem + animal/primate imaging	Compensatory DAT upregulation / altered transport function with chronic exposure	**Moderate**	Heterogeneity in exposure; post-mortem selection; translation to clinical progression	Little et al., 1998; Mash et al., 2002; Letchworth et al., 2001
**Fronto–striatal structural changes**	Structural MRI (human)	Abnormal frontostriatal morphology; PFC/striatal alterations linked to impulsivity/compulsivity	**Moderate**	Vulnerability vs exposure effect; age/vascular risk; sample selection	Ersche et al., 2011; Ersche et al., 2012; Narayana et al., 2010
**Gray/white matter patterns at scale**	Large-cohort / meta-analytic neuroimaging	Common + sex-specific associations; heterogeneous but reproducible patterns	**Moderate**	Polysubstance, psychiatric comorbidity; site/scanner variability; exposure quantification	Rabin et al., 2022; Dang et al., 2022 meta-analysis; Ceceli et al., 2023
**White-matter integrity**	DTI (human)	Reduced FA / altered diffusivity in callosal/frontal/association tracts	**Moderate**	Levamisole/alcohol; TBI; vascular/metabolic risk; abstinence duration	Xu et al., 2010; He et al., 2020; Michels et al., 2022
**Network dysregulation**(DMN/SN/CEN)	Resting-state fMRI (human)	Disrupted SN–DMN interactions; network dysregulation linked to severity/outcome	**Moderate**	State effects (withdrawal/recent use), analytic variability, motion	Liang et al., 2015; Geng et al., 2017; Worhunsky et al., 2013
**Perfusion / glucose metabolism**("hypofrontality")	FDG-PET / perfusion studies	Reduced frontal/cingulate metabolism persisting in withdrawal/abstinence	**Moderate**	Vascular factors; medications; comorbid depression; small samples	Volkow et al., 1991; Volkow et al., 1992; Lane et al., 2010
**Oxidative stress & mitochondrial dysfunction**	Mainly preclinical + cellular + emerging translational	ROS increase; mitochondrial dynamics impairment; neuronal vulnerability	**Moderate (translational)**	Translation gap animal→human; regimen differences; lack of longitudinal human biomarkers	Poon et al., 2007; Wen et al., 2022; Cole et al., 2021
**Neuroinflammation / microglial activation / BBB-neurovascular axis**	Preclinical + narrative/translational	Microglial activation; neurovascular/BBB vulnerability as permissive mechanism	**Emerging**	Indirect markers; strong confounding by HIV/infections & systemic inflammation	da Silva et al., 2023; López-Pedrajas et al., 2015; Clare et al., 2024
**Protein handling signals** (α-synuclein)	Post-mortem + translational/preclinical	Increased α-synuclein expression signals in cocaine abusers	**Emerging / preliminary**	Post-mortem selection; causality unclear; not proof of synucleinopathy progression	Mash et al., 2003; Qin et al., 2005; Mash et al., 2008

**VMAT2 vulnerability hypothesis**(dopaminergic resilience)	Mechanistic / disease-model literature	Reduced vesicular DA storage increases oxidative vulnerability (conceptual bridge)	**Preliminary (conceptual)**	Not directly demonstrated in CUD in vivo; inferential linking	Eiden & Weihe, 2011; Caudle et al., 2007; Chen et al., 2008
**Cerebellar involvement**(structure/learning)	Human MRI + targeted cognitive associations + reviews	Cerebellar GM reductions and learning deficits associated with severity	**Emerging**	Smaller cohorts; heterogeneity; vascular/metabolic confounds; polysubstance	Sim et al., 2007; Moreno-López et al., 2015; Miquel et al., 2020
**Clinical motor abnormalities / movement disorders**	Narrative review + clinical literature	Wide spectrum (parkinsonism, choreoathetosis, cerebellar signs)	**Hypothesis-generating**	Differential diagnosis (vascular events, toxins, comorbidity); case-selection	Byroju et al., 2024 review; Cenci et al., 2022; Toossi et al., 2010
**Reversibility with abstinence** (cross-domain)	Mixed; some longitudinal/treatment-linked imaging	Partial recovery in subsets vs persistent alterations in others	**Uncertain (key divergence)**	Abstinence duration/quality; treatment exposure; baseline vulnerability; timing of assessments	He et al., 2020; McCurdy et al., 2024; Xu et al., 2010
